# Engineering bone-marrow in a dish – a bloody business: Pre-clinical opportunities, translational use-cases, and a call for consensus

**DOI:** 10.1016/j.jtha.2026.02.018

**Published:** 2026-03-04

**Authors:** Daniel North, Grace A. Meaker, Abdullah O. Khan

**Affiliations:** 1 Faculty of Medicine, Dentistry and Health Sciences, University of Melbourne, Melbourne, VIC, Australia; 2 ACRF Translational Research Laboratory, The Royal Melbourne Hospital, Melbourne, VIC, Australia; 3 MRC Molecular Haematology Unit, Weatherall Institute of Molecular Medicine, Radcliffe Department of Medicine and National Institute of Health Research (NIHR). Oxford Biomedical Research Centre, University of Oxford, Oxford, United Kingdom

**Keywords:** Organoids, bone marrow, tissue engineering

## Abstract

The bone marrow is the principal site of blood and immune cell production throughout the adult lifespan. Changes within bone marrow niches which regulate haematopoiesis directly contribute to age-related immune decline, an increased risk of cancer and cardiovascular disease, and the onset and progression of blood cancers. Targeting bone marrow dysfunction remains a major translational challenge, where species differences limit the predictive power of animal models and current in vitro systems fail to capture the cellular and architectural complexity that enables lifelong haematopoiesis. The pressing need in the field is for human alternatives. These should accelerate basic discovery (where direct experimentation on living marrow is neither ethical nor practical), personalised medicine (where efficacy and toxicity could be tested in patient relevant models to guide treatment decisions), and preclinical drug development. Advances in stem cell engineering, biomaterials, and micro-physiological systems have brought the idea of building a human bone-marrow niche ex vivo closer to experimental reality. In this review, we examine recent advances in bone marrow organoid development through the lens of how these systems can meet translational needs. We propose that to coordinate progress across the field, there is a need to develop consensus benchmarks to define an ideal bone marrow organoid that can overcome translational barriers. We suggest that a bone marrow organoid should (1) reproduce the diverse stromal and haematopoietic lineages of the bone marrow, (2) self-organize into spatial architectures that recapitulate human haematopoietic niches, and (3) sustain lympho-myeloid output from progenitors over time.

## Introduction

The bone marrow is the primary site of haematopoiesis after birth, sustaining the production of blood and immune cells throughout life (1,2). Haematopoietic stem cells (HSCs) reside at the apex of the haematopoietic hierarchy and are uniquely capable of indefinite self-renewal and multi-lineage differentiation.

The adult marrow is compartmentalised into microanatomical ‘niches’ invested with specialised stromal and endothelial cells (3,4). HSC localisation and quiescence are dependent on the production of growth factors such as SCF and CXCL12 by Leptin-receptor^+^ mesenchymal stem cells (MSCs) (5,6). Osteolineage cells and osteoblasts populate endosteal regions, where osteoblast-derived NOTCH ligands are one of many key factors influencing early lymphopoiesis and HSC fate decisions (7,8). The bone marrow niche integrates biochemical, signalling, and oxygen gradients which regulate haematopoiesis in health and disease (1,3).

Studying human bone marrow remains technically challenging. The dense, calcified architecture of bone impedes high-resolution imaging and spatial omics, though recent studies are beginning to resolve cellular topology at unprecedented detail (9,10). Sampling is invasive and spatially limited, restricting systematic analysis of marrow heterogeneity (11). Moreover, “healthy” controls often derive from older or clinically investigated donors, confounding interpretation (12,13).

As a result, much of our understanding derives from murine models. While indispensable as ‘complete’ experimental models, mouse and human marrows differ in structure, cellular composition, and ageing trajectories (1,3). Murine bone marrow fails to capture key human features such as prominent age-related bone marrow adipogenesis, a loss of clonal diversity and the emergence of clonal haematopoiesis, processes central to haematopoietic ageing and the pathogenesis of myeloid malignancies in humans (14,15).

The convergence of induced pluripotent stem-cell (hiPSC) technology, biomaterials science, and micro-physiological “organ-on-chip” systems has transformed the feasibility of reconstructing the marrow niche in vitro (16–22). Early three-dimensional (3D) models relied on scaffolds seeded with primary stromal and haematopoietic cells, or vascularised hydrogel matrices populated with endothelial cells (3,23–26). While these approaches provided proof-of-concept, most employed simplified components—such as human umbilical vein endothelial cells (HUVECs) instead of bona fide sinusoidal endothelium—or lacked the stromal diversity necessary to maintain functional haematopoiesis. Many were also technically complex, and challenging to reproduce, restricting their adoption beyond specialist laboratories.

The emergence of organoid technologies—self-organising, multicellular 3D cultures derived from human tissue or pluripotent stem cells—has revolutionised tissue modelling (27,28). In the bone-marrow field, organoid and “assembloid” approaches aim to capture the intrinsic capacity of stem-cell populations to generate organised, vascular microenvironments functionally capable of supporting haematopoiesis (17). These systems offer a uniquely human platform to dissect developmental hierarchies, disease mechanisms, and drug responses in a physiologically relevant context.

**Figure 1:**
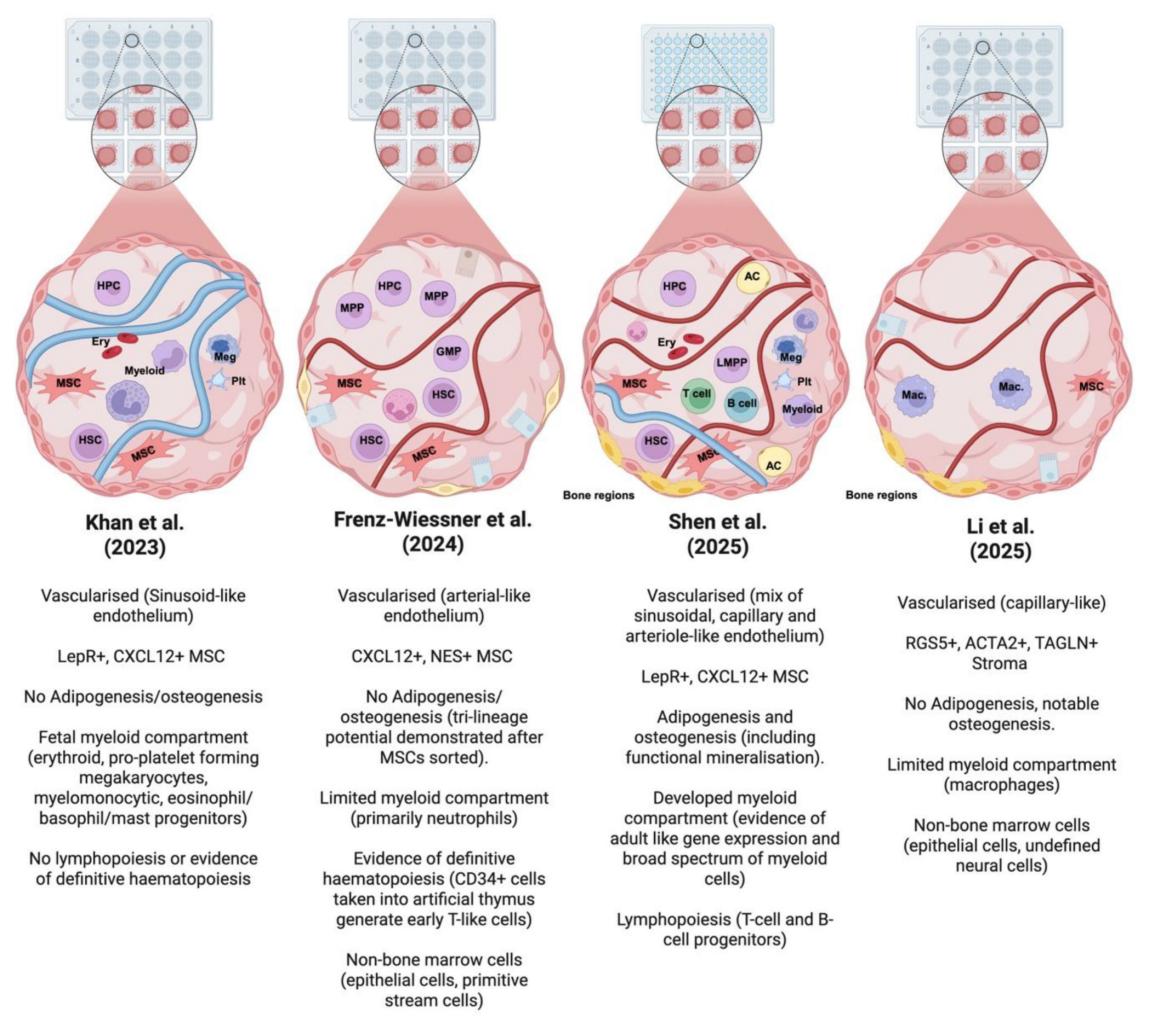
Simplified graphical comparison between four current organoid models. A comparison of the 4 reported bone marrow organoid (BMO) systems published thus far: Khan *et al*. (2023), Frenz-Weissner *et al*. (2024), Li *et al*. (2025), Shen *et al*. (2025, *Cell Stem Cell, in press*). All three employhiPSCs in a 3D scaffold to generate a self organized vascular structure. Shen et al. use a granular hydrogel to improve scalability and throughput. HSC = haematopoietic stem cell, HPC = haematopoietic progenitor cell, MSC = mesenchymal stromal cell, Ery = erythrocyte, Meg = megakaryocyte, Plt = platelet, LMPP = lymphoid-primed multipotent progenitor cell, AC = adipocyte, MPP = multipotent progenitor cell. Created in https://BioRender.com.

Paul Bourgine’s recent review comprehensively catalogued technical advances in this space and compared the major bone-marrow organoid (BMO) models, including those by Frenz-Weissner et al. (Nature Methods 2024), Khan et al. (Cancer Discovery 2023), and Shen et al. (preprint 2024)(29). Since this publication, Li *et al*. have reported the generation of an engineered vascularized osteoblastic niche (eVON) (30). These four organoid models are graphically summarised in Figure 1. In his review, as in ours, we focus on organoids defined as ‘self-organising, vascular 3D cultures comprising both stromal and haematopoietic cells’.

Rather than re-iterate that analysis, our review focuses on the translational promise of BMOs—how these systems can bridge experimental models and clinical application, inform therapeutic design, and serve as platforms for precision medicine. We discuss their potential to replace or complement animal models in pre-clinical research, outline key biological and technical challenges, and propose consensus benchmarks to define an *ideal* bone-marrow organoid to help address key translational challenges.

### Background: Mice & Men - Human tissue to study human haematopoiesis

Our understanding of the organisation of the haematopoietic niche has relied heavily on animal models—particularly mice—to dissect the structure and function of the bone marrow (1,3). Three-dimensional imaging and omics modalities have since revealed an intricate architecture, but major species differences are increasingly evident. This section highlights translationally important differences requiring human-specific models.

While mice and humans share core principles of HSC regulation, key disparities in niche composition, stromal function, and ageing trajectories limit the translational fidelity of murine models (31). Although both species show reduced HSC quiescence and lymphoid output with age, in ageing human bone marrow demonstrates marked adiposity and a reduced diversity of the clonal HSC pool (13–15,32). Table 1 summarises selected differences between human and mouse haematopoiesis, highlighting slower HSC cycling, greater adiposity, and a higher baseline myeloid fraction in humans.

A further translational gap arises from the phenomenon of **clonal haematopoiesis** (15). Clonal haematopoiesis of indeterminate potential (CHIP) has emerged as one of the most significant human-specific features of marrow ageing, with profound clinical implications. Somatic mutations in epigenetic regulators such as *DNMT3A, TET2*, and *ASXL1* confer a competitive advantage to HSCs, driving clonal expansion of mutant progenitors (40,41). Recent work by Kapadia *et al*. characterised the acquisition of somatic mutations in murine stem cells and progenitors, reporting a threefold greater rate of mutation in mice (15). Despite this, ageing mice do not appear to demonstrate the loss of clonal diversity that is a hallmark of human ageing, nor do they demonstrate the total mutational burden observed in humans (likely a consequence of vastly different lifespans) (15). CHIP-like clonal expansions can occur in mice under stress or with particular engineered drivers, but are not observed in physiological ageing to the same extent or penetrance as in humans (42,43).

CHIP increases the lifetime risk of haematologic malignancies and is independently associated with a two- to fourfold higher incidence of atherosclerotic cardiovascular disease and heart failure, largely through mutant myeloid–driven inflammation (40). Cytotoxic therapy and chronic inflammation further accelerate clonal selection, making CHIP a plausible driver of therapy-related myeloid neoplasms and relapse (44,45).

Human haematopoietic stem cells acquire roughly 17 somatic mutations per year, whereas murine HSCs accumulate ∼45, but rarely undergo clonal outgrowth (15,32,46). This may reflect the short lifespans, and pathogen-controlled environments of laboratory mice, which limit the selective pressures driving CHIP in humans (47). Efforts to model CHIP through inflammatory or mutagenic stimulation only partially recapitulate human phenotypes (15).

BMOs capable of maintaining long-term haematopoiesis could offer an opportunity to model these processes. Longitudinal single-cell sequencing in stable 3D cultures can track clonal trajectories and define the microenvironmental cues which govern clonal fitness. To date, the only longitudinal sequencing performed to our knowledge was reported by Shen *et al. (10x RNA sequencing)*, where maturation and a shift from a fetal to adult phenotype is observed when comparing samples sequenced at days 20 and 35 respectively (48).

Although current-generation BMOs are cultured for weeks, advances in matrix design, vascularisation, and the implementation of dynamic flow may bring long-term, functionally stable systems capable of sustaining months of haematopoiesis. Thus far no organoid has demonstrated a long-term stable culture capable of modelling clonal phenomenon like CHIP.

### Preclinical Modelling 1: Malignancy in context

The role of the niche in the onset, progression, and relapse of haematological malignancies has taken centre stage in recent years (3). We will discuss cancer-niche interactions in myeloproliferative neoplasms (MPNs) and multiple myeloma as representative examples of myeloid and lymphoid malignancies respectively with major unmet clinical needs (49,50). They are examples of malignancies which cannot be understood abstracted from the tissue context of their tumour micro-environment, a context which existing murine models incompletely recreate. BMOs can be tractable systems to uncover niche-dependent vulnerabilities in these malignancies. We will summarise publications where BMOs have been employed in this capacity, as well as highlighting opportunities where we hypothesise these systems could be used in the future.

MPNs arise from an HSC which has acquired a driver mutation, providing a competitive advantage leading to the gradual expansion of the malignant clone (51). MPN cells then co-opt the local bone marrow microenvironment to support clonal expansion at the expense of normal haematopoiesis (52).

In Philadelphia-negative MPNs, the *JAK2*^*V617F*^ mutation resulting in constitutive activation of the JAK2 tyrosine kinase is the most common oncogenic driver (53). Distinct HSC-niche interactions have been identified in different *JAK2*^V617F +^ MPNs, highlighting the role of the bone marrow microenvironment in shaping disease phenotypes (54). For example, in essential thrombocythemia (ET) HSCs localised to an endosteal, peri-arteriolar niche, while in polycythemia vera (PV) HSCs localised to a central marrow, perisinusoidal niche (54).

Studying the relationships between MPN cells and niche stromal populations can identify novel therapeutic targets. Our colleagues recently demonstrated that galactin-1 and its receptor b1-integrin mediate communication between malignant haematopoiesis and key niche stromal populations in MPNs (55). Inhibition of galactin-1 reduced TGF-b induced fibrosis in bone marrow organoids and ameliorated the disease phenotype in a murine model of primary myelofibrosis (PMF). The Schneider group have recently identified S100A8/A9 alarmin signalling as lynch pin of a bidirectional axis between malignant haematopoiesis and bone marrow MSCs (56). S100A8/A9 was upregulated in MSCs in both murine models of MPN and PMF patient samples, where S100A8 expression was also predictive of disease progression (57). This axis is targetable, with the S100A8/A9 inhibitor tasquinimod preventing fibrosis and improving haematopoietic parameters in murine models of MPN. Excitingly, tasquinimod has entered a phase Ib/II clinical trial (clincaltrials.gov ID: NCT06605586) for the treatment of PMF, highlighting the translational potential of studying interactions between haematological malignancies and the niche.

Murine models of MPN have provided insights into these conditions but fail to capture important elements of disease (58). An unanswered question in MPN biology is how the same genetic lesion can result in diverse clinical phenotypes, with the *JAK2*^*V617F*^ mutation acting as the driver in the majority of patients with PV, ET and PMF (59). Patient derived xenografts (PDX)s from *JAK2*^*V617F*^ PV and ET patients localise to niches in the marrow in a manner analogous to the differential distribution of HSCs in bone marrow biopsies taken from PV and ET patients, but fail to recapitulate other hallmarks of these conditions (54,60). Controlling the dosage of *JAK2*^*V617F*^ in transgenic mice by placing its expression under the control of different promoters can generate PV-like and ET-like models (61).

Another limitation in current models of MPN is the lack of representative murine models of PMF. One of the most broadly used models of PMF involves the transduction of the *MPL*^*W515L*^ into murine HSCs, prior to their transplantation into irradiated wild-type recipients (62). This results in a rapidly progressive PMF-like MPN syndrome characterized by splenomegaly, anaemia and reticulin fibrosis leading to death within four weeks of transplantation. The rapid pace of disease progression is out of keeping with the phenotype seen in human *MPL*^*W515L*^ driven MPNs, including PMF. PDX models of PMF have been historically challenging due to poor xenotransplantation potential of patient HSCs (63). Current generations of bone marrow organoids support the engraftment of unmanipulated PMF patient HSCs, resulting in characteristic reticulin fibrosis and niche remodelling (64). This approach enables scalable pre-clinical testing.

Generating material from patient-derived hiPSC lines could facilitate a scalable and genetically controlled interrogation of interactions between mutant haematopoietic clones and the bone marrow microenvironment. Haematopoietic cells differentiated from patient derived hiPSC lines retaining relevant malignancy-associated somatic mutations have been shown to model disease characteristics in MPNs and AML (65–68). MPN patient peripheral blood CD34^+^ cells would be reprogrammed into hiPSCs, and then could be reproducibly differentiated into MPN-like CD34^+^ haematopoietic progenitors using recently described iHSC differentiation strategies (134). These mutant CD34^+^ cells could be seeded into BMOs derived from isogenic, mutation-corrected or non-haematopoietically derived patient hiPSC lines to isolate cell-intrinsic versus niche-mediated disease effects.

Multiple myeloma is a malignancy of plasma cells (PC), terminally differentiated B-cells primarily located in the bone marrow (69). Under physiological conditions, PC retention survival is dependent on extrinsic factors produced by stromal and myeloid cells within the PC niche, including CXCL12, APRIL and IL6 (70–72). This dependence on niche support persists in the malignant PCs of myeloma (73,74), where inflammatory MSCs (75) and neutrophils are now known to be key drivers (76).

MSCs harvested from myeloma patients are more capable of supporting PCs ex vivo than MSCs from healthy controls (77,78). Recent studies incorporating single-cell and spatial transcriptomics have identified that highly heterogenous, spatially segregated populations of both plasma cells and peri-tumour stroma exist even within individual plasmacytomas (79,80).

Myeloma cells are sufficiently dependent on the bone marrow niche that most primary cells are unable to survive and expand ex vivo. Many of the cell lines in common use have been derived from rare patients with circulating or extra-medullary disease (82). A strength of BMOs for the study of myeloma is the simple fact that they reliably facilitate the engraftment and expansion of primary myeloma cells ex vivo, which overcomes a significant technical challenge in the field (64).

Most current murine models of myeloma do not facilitate the study of interactions between myeloma cells and the bone marrow niche (83,84). The observation that in aging, C57BL/KaLwRij mice have a propensity to spontaneously develop a monoclonal gammopathy with rare progression to a myeloma-like malignancy led to the development of the 5TMM model (85). Cell lines derived from these murine cancers readily engraft in syngeneic wildtype mice and recreate many hallmarks of human myeloma.

The strength of these models is that they allow myeloma to be modelled in immunocompetent mice, but while myeloma-like, this murine malignancy is fundamentally a biologically distinct entity. For example, 5TMM plasma cells do not show evidence of somatic hypermutation, and rarely involve alterations of c-myc, a common lesion in human myeloma (86).

Most patient derived xenograft (PDX) models in myeloma suffer from the same limitation as studying myeloma cells in vitro; the dependency of myeloma cells on the human marrow niche precludes most primary patient samples and requires the use of specific cells lines with a capacity for extramedullary expansion (83). A recent advance has been the development of MIS^(KI)^TRG6 mice, which express human M-CSF, IL-3, GM-CSF, TPO, SIRP_a_ and crucially IL6 (87). This model reproducibly supports the engraftment of primary patient samples, an important milestone.

There is still a need to understand the interactions of myeloma with human stromal populations in the bone marrow niche. Previous approaches have involved implanting immunodeficient mice with human foetal bone chips, or synthetic bone scaffolds seeded with human MSCs (88,89).

### Preclinical Modelling 2: Drug efficacy and resistance in a tissue context

The success or failure of treatments for haematological cancers can also be dictated by complex interactions within the bone marrow niche. We will use the immunomodulatory drugs (IMiDs®) in myeloma and T-cell-based therapeutics as representative examples to highlight the shortfalls of performing pre-clinical drug development in systems which abstract drug-target interactions away from a clinically relevant tissue context.

The thalidomide derived IMiDs form a cornerstone of myeloma treatment, and exhibit both tumor-intrinsic and extrinsic mechanisms underpinning efficacy and treatment failure (90). IMiDs act via binding to cereblon (CRBN), an E3 ubiquitin-ligase adaptor leading to the ubiquitin mediated degradation of IKZF1 and IKZF3, transcription factors myeloma depends on (91,92). A less well understood, but crucial contribution to the action of IMiDs is modulation of the local immune microenvironment (93). IMiDs have been shown to enhance the function and cytotoxicity of T and NK cells both in in vitro models and in patients (94–97). IMiDs also undermine the support of malignant PCs by niche stromal populations by downregulating the adhesion molecules that facilitate these interactions.

The best characterized mechanisms of IMiD treatment failure are acquired mutations in CRBN or its binding partners within malignant PCs (98–100). Considering IMiD resistance in a tissue context also reveals targetable tumor-extrinsic pathways. The adhesion molecule CD44 is enriched on myeloma cells from lenalidomide refractory patients, and results in enhanced adhesion to niche stromal populations via binding by its ligand hyaluronic acid (HA) (101). Disrupting CD44-HA interactions resulted in re-sensitization to lenalidomide both in vitro and in murine models.

In keeping with the role of T-cell activation in the efficacy of IMiDs, T-cell exhaustion in the bone marrow microenvironment is a consistent feature of relapsed and refractory myeloma (48–50)reference error. Understanding the mechanisms of T-cell exhaustion in haematological malignancies is of increasing importance, as therapies such as chimeric antigen receptor T-cells (CAR-T)s and bi-specific T-cell engagers (BiTE)s become increasingly prevalent (102). T-cell exhaustion is also emerging as an important cause of CAR-T treatment failure across disease subtypes (103,104). An immunosuppressive local microenvironment undermining T-cell fitness and persistence is a major barrier to the success of cellular therapies in solid tumours (105).

Co-culture experiments have demonstrated that the addition of MSCs inhibits the ability of CAR-T-cells to kill lymphoma, and results in loss of cytotoxic CD8^+^ T-cells and an increase in immune suppressive T_reg_ cells (106). Similar experiments have demonstrated that patient-derived MSCs also impair tumour killing by CAR-T cells in myeloma, in part by upregulating the intrinsic anti-apoptotic machinery of malignant PCs (107). Sakemura and colleagues overcame stromal-mediated inhibition of CAR-T function in myeloma by developing CAR-T cells with dual specificity for BCMA expressing PCs, and FAP/SLAMF7 expressing cancer-associated fibroblasts (108).

The need for model systems to study cellular therapeutics in a relevant tissue context is already being filled by a new generation of 3D culture systems. The Roy group have recently described a multi-niche marrow-on-a-chip system for evaluating interactions between myeloma, CAR-T-cells and the marrow microenvironment (109). We have recently presented work using bone marrow organoids engrafted with CD34+ cells from MPN patients for the preclinical validation of a first-in-class anti-mutant calreticulin (mutCALR) CAR-T for the treatment of mutCALR+ MPNs (110). An open question remains about the degree and role of allogeneic response where there is a donor-organoid mismatch, and future work will need to dissect this.

Tractable model systems that allow therapeutics to be evaluated in a relevant tissue context will facilitate rationale interventions to improve the safety and efficacy of treatments that are working, and to circumvent failures due to niche-mediated drug resistance (111).

### Preclinical Modelling 3: Disorders of haemostasis and thrombosis in a tissue context

The need to consider disorders of the haematopoietic system in a tissue context extends beyond malignancy. Immune thrombocytopenic purpura (ITP) is a clear example. ITP is an autoimmune condition characterized by persistent thrombocytopenia, in part due to antibody-mediated peripheral platelet clearance (112). In addition to auto-antibody production, the pathophysiology of ITP involves dysregulation of T-cell, NK cell and dendritic cell populations (113). ITP is not only caused by increased peripheral platelet turnover but involves immune-mediated functional deficits of megakaryocytes (114–116).

Thrombopoiesis is regulated by interactions with the stromal populations which define the bone marrow niche (117). It is likely that changes in stromal populations contribute to the immune dysregulation which drives ITP. MSCs collected from patients with ITP compared with healthy controls have an impaired capacity to regulate T-cell activation and less efficiently support the differentiation of immunomodulatory T-cell populations (118–121). Co-culture of MSCs from healthy donors with dendritic cells (DCs) induces a tolerogenic DC phenotype capable of suppressing CD4^+^ differentiation into Th_1_ cells implicated in ITP (113). In contrast, MSCs from ITP patients have a markedly reduced capacity to promote tolerogenic DCs (122).

ITP is a clear example of a condition which would benefit from model systems incorporating human haematopoiesis, bone marrow stroma and immune populations. Alessandra Balduini’s group employed a novel 3D tissue model of human bone marrow to explore TPO agonist responses in inherited thrombocytopenia syndromes, highlighting the value of these approaches in the study of disorders of platelet biology (123).

### Bone marrow organoids for precision medicine

**Figure 2:**
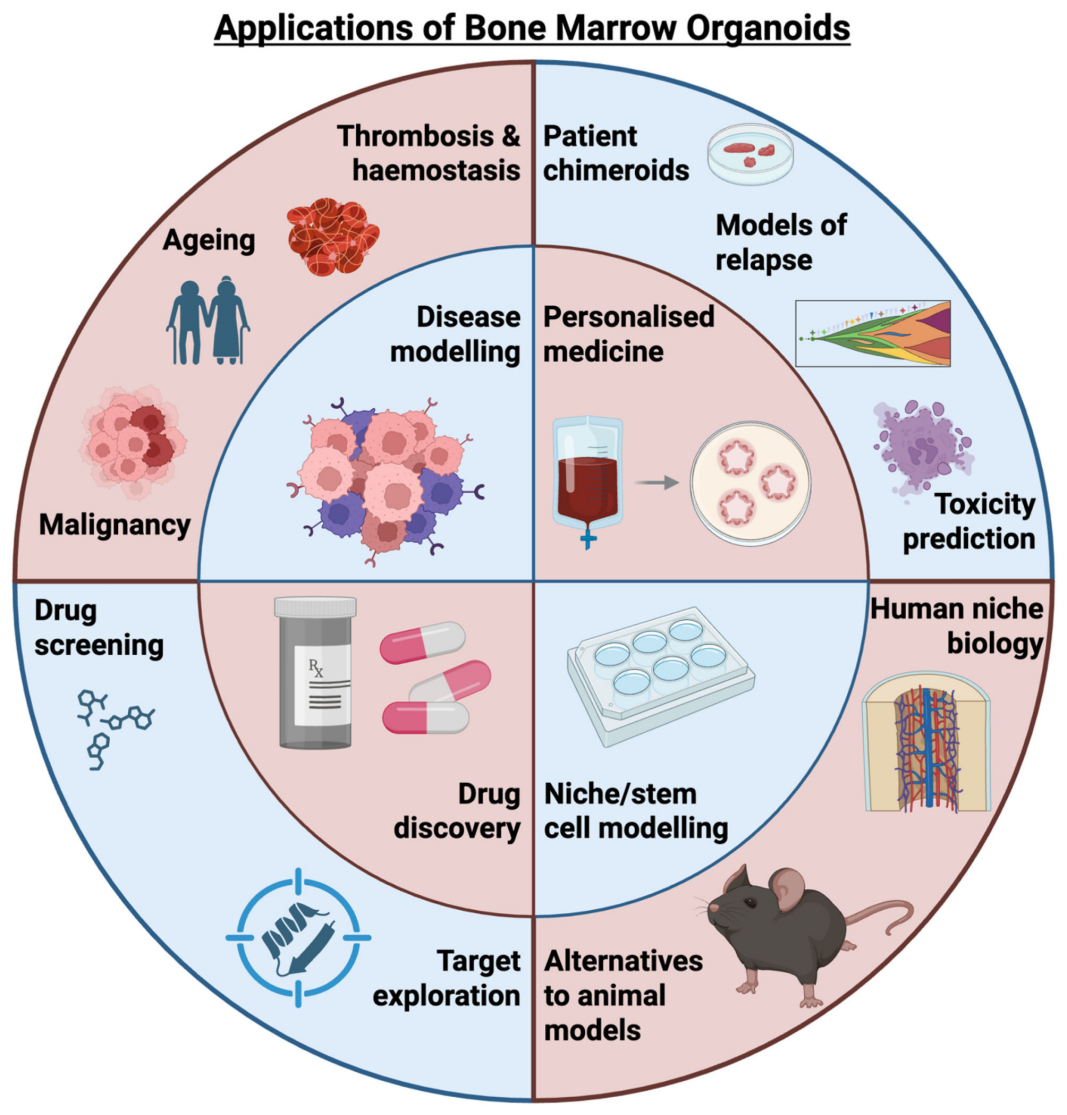
Clinical and biological applications of bone marrow organoids. The major applications highlighted by recent work have been in disease modelling (malignancy, ageing, and thrombosis and haemostasis), the generation of patient chimeroids (work by Khan *et al*., Ren *et al*.,Shen *et al*. demonstrate that bone marrow organoids can support the growth and expansion of patient cells from multiple myeloid and lymphoid cancers). As demonstrated by other organoid fields, in vitro models have the potential to reveal new targets and therapies – though the bone marrow organoid field remains nascent in this regard. BMOs offer a viable alternative to animal models in specific instances, ranging from fundamental research into the biology of haematopoiesis, through to modelling haematological malignancies or disorders of thrombosis and haemostasis. As a tool for personalised medicine, BMOs have demonstrated potential as tools to predict safety and efficacy, but further work will be needed to establish the translational potential of these model systems, particularly in the context of refractory disease Created in https://BioRender.com

The promise of organoid technologies as platforms for precision medicine is enormous, but they are only now beginning to translate into predictive technologies delivering impact at the patient bedside. The appeal of the concept is obvious: derive a physiologically relevant model system incorporating patient tissue, trial a proposed treatment in this system, and draw insights regarding efficacy or toxicity. Applications of bone marrow organoids, both current and future, are summarised in Figure 2.

While BMOs remain an emerging field, epithelial organoids as a platform for precision medicine are a more mature technology (124). A landmark study using organoids derived from gastrointestinal cancers in 21 patients demonstrated that ex vivo chemo-sensitivity studies in organoids had an 88% positive-predictive value and 100% negative-predictive value for treatment responses in the patients from whom the organoids were derived (28). While small in scale, this established organoids as a viable predictive platform and led the way for studies using organoids as a high-throughput system to screen for chemotherapy sensitivity and guide treatment decisions in solid malignancies refractory to multiple prior lines of therapy.

The APOLLO study used peritoneal organoids derived from patients with unresectable peritoneal metastases of colorectal cancer to guide chemotherapy prescribing, leading to changes in treatment in two patients and to a clinically significant response in one patient (125).

Jensen and colleagues derived organoids from tumour samples in 82 patients with relapsed/refractory colorectal cancer, leading to organoid directed treatment in 34, with 17 meeting the pre-specified primary endpoint of progression free survival at 2 months (126). Excitingly, a larger scale phase III trial randomising patients with treatment refractory breast cancer to either receive physicians’ choice of treatment or organoid guided therapy is currently recruiting (clinicaltrials.gov ID: NCT06268652) This trial will be using a previously published platform employing patient-derived organoids to predict chemo-sensitivity in breast cancer (127).

Recent publications demonstrate that BMOs have achieved an important proof of principle: BMOs can meaningfully recreate features of human disease. Our group has previously demonstrated that organoids engrafted with CD34^+^ cells from myelofibrosis patients, but not healthy controls, demonstrate fibrosis and niche-remodelling (64). The VAF of primary patient samples was maintained over the course of a 2-week culture within the organoid, and a pilot proof-of-concept experiment assessing the utility of this platform to address drug efficacy was performed (64). We showed that organoid engraftment with multiple donor derived cells from patients with multiple myeloma (n = 5), acute lymphoblastic leukaemic (ALL), and a xenograft model of infant ALL (iALL) demonstrated consistent growth and expansion over 2-weeks, consistently outperforming primary bone marrow MSC co-culture (64). In related work, Ren et al. utilised the same BMO model and demonstrated that engraftment studies could also be performed using primary samples from patients with myelodysplastic syndrome (128).

More recently we have built on this work in the recent pre-print reporting the generation of a combined bone and bone marrow organoid (comBO) (48). This is the first organoid model to replicate an osteoblastic niche as well as lympho-myeloid differentiation, vascularisation, and adipocyte formation within a single hiPSC derived system. Primary myeloma cells significantly remodelled engrafted BMOs, reproducing the pro-inflammatory changes in stromal populations which have been identified as drivers of progression, relapse and therapeutic resistance in the myeloma microenvironment (12).

Frenz-Weissner and colleagues developed organoids from hiPSCs carrying the *VSP45*^*T224N*^ mutation associated with *VSP45* deficiency, a bone marrow failure syndrome characterised by neutropenia and myelofibrosis (129). These organoids demonstrated increased reticulin fibrosis, an expansion of myofibroblast-like stromal cells, and increased neutrophil apoptosis.

The next step towards BMO guided precision medicine will be to demonstrate that these platforms have a capacity to model not only pathophysiology in general, but to recreate features specific to individual patients. While recent publications begin to hint at this result, we believe that reproducible and quantitative validation of this supposition is urgently required (128,129).

Platforms for precision medicine also need to demonstrate that they are predictive of treatment efficacy or toxicity at an individual patient level, and these experiments have not yet been performed in BMOs. The impact of chemotherapy and radiation on stromal and haematopoietic populations have been explored in 3D-culture systems seeded with patient-cells (130,131).

### Defining targets and benchmarks for the field: the case for consensus

A central unresolved issue in tissue engineering is *definition*—where along the continuum of complexity and function does a construct qualify as a bona fide organoid?

In 2014, Lancaster and Knoblich proposed three minimal criteria to define an organoid: (1) the presence of multiple organ-specific cell types; (2) the ability to recapitulate characteristic organ functions; and (3) spatial organisation resembling the native tissue (132). The neuro-organoid field rose to the challenge of defining consensus criteria and benefited from a structured debate on minimal requirements and shared standards. As the field of BMOs evolves, an equivalent conversation is urgently needed: what would constitute an *ideal* BMO that meaningfully addresses translational barriers? In which contexts could these systems replace animal models?

Here, we adapt the framework proposed by Lancaster and Knoblich to the specific biology of the bone marrow, recognising that this tissue is a dynamic and regenerative system rather than a morphogenetic organ. We discuss three inter-related criteria—cellular diversity, sustained function, and spatial organisation—as guideposts toward consensus.

#### 1 Cellular diversity and lineage breadth

Lifelong haematopoiesis depends on a complex interplay between haematopoietic stem and progenitor cells (HSPCs) and a diverse stromal microenvironment that includes sinusoidal and arteriolar endothelial cells, mesenchymal stromal populations such as LepR^+^ MSCs, osteolineage cells, and marrow adipocytes. These elements together support balanced lymphoid and myeloid output, regulate quiescence, and maintain immune competence. An organoid claiming to represent bone marrow should therefore reproduce not only haematopoietic but also stromal and vascular heterogeneity in appropriate proportions. Bone marrow specific cell types known to be essential to bone marrow function should be incorporated, e.g. sinusoidal endothelium, LepR+ MSCs, and osteoblasts.

Rigorous benchmarking of organoid transcriptomes to primary tissues remains essential. Single-cell atlases of adult and fetal haematopoiesis now provide reference maps for such comparisons, though results depend strongly on dataset quality and computational integration (133).

#### 2 Sustained haematopoietic function

Functional validation will be the most important metric of organoid fidelity. The bone marrow’s defining physiological role is the lifelong maintenance of haematopoiesis. By this logic, the minimal functional benchmark for a BMO should be the ability to sustain multilineage haematopoiesis—either from endogenously generated or donor-derived progenitors—*without* exogenous cytokine supplementation other than thrombopoietin (TPO) and erythropoietin (EPO), which physiologically originate in other organs (134–137). Sustained equilibrium of stem and progenitor populations, rather than transient output, would verify durable haematopoietic support and the presence of self-renewing HSCs (138).

Ng and colleagues developed induced HSC-like cells (iHSCs) from human hiPSCs that supported long-term, multilineage engraftment in immunocompromised mice, even after cryopreservation (139). This milestone, once considered unattainable, provides a functional yardstick for in vitro haematopoiesis.

A logical next step is the development of serial organoid re-engraftment assays—an in vitro analogue of murine serial transplantation—to test self-renewal capacity. In this paradigm, organoids are engrafted with CD34^+^ cells (either organoid- or donor-derived), their output tracked over time, then dissociated and re-engrafted into fresh scaffolds. Maintenance of CD34^+^ fractions and restoration of lineage diversity across passages would provide a stringent in vitro measure of self-renewal without cytokine supplementation. The timescale of these assays require consideration. By convention, sustained multilineage output at a 16-week timepoint post-transplant is considered evidence of the output of long-term HSCs in murine models, though it has long been recognised that rare cells without a capacity for indefinite renewal can persist beyond 16 weeks (138,140). Recently, Shen *et al*. demonstrated a capacity for serial organoid re-seeding using both donor derived CD34+ cells and comBO derived CD34+ cells. While this work did not thoroughly investigate the phenotype of serially re-transplanted CD34+ cells in this context, it did show that after 3 successive 2-week transplants, both primary donor and comBO derived CD34+ cells retained lympho-myeloid potential.

While such assays cannot replace in vivo transplantation as the definitive test of HSC potency, they represent a feasible intermediate benchmark toward demonstrating bona fide repopulating potential. In the context of the study of malignancy, systems that enable a long-term culture that facilitates modelling refractory disease will be essential as relapse remains a major clinical obstacle.

#### 3 Spatial organisation and benchmarking to native tissue

Benchmarking the spatial architecture of BMOs is impeded by our incomplete understanding of bone marrow microanatomy. Even within the murine literature, controversy exists regarding the organisation of functionally distinct micro-niches. The majority of cycling HSCs exist in a central-perisinusoidal niche (141), while a functionally distinct periarteriolar niche has been identified as enriched for quiescent HSCs, but leading groups have argued against the relevance of this compartment (3,142). Comparative analyses of recent transcriptomic atlases of murine and human marrow have revealed both conserved and divergent features, raising questions about the generalisability of murine models to the human niche (9,143,144). “Ground-truth” regarding the spatial organisation human marrow will continue to evolve, and BMO benchmarks will need to evolve in step.

The long-term vision for BMOs extends beyond discovery-science and pre-clinical drug testing to patient-specific precision medicine. Patient-derived “chimeroids” could in principle predict response or resistance to therapy and guide individualised dose selection. Realising this ambition will require models capable of therapeutic response assessments which can be prospectively benchmarked against parallel clinical patient outcomes, mirroring approaches validated in gastrointestinal tumour organoids (125,145).

### Concluding remarks

In vitro bone-marrow modelling has advanced at an extraordinary pace, the next frontier will be to formalise a consensus functional definition of a bone-marrow organoid—anchored in sustained multilineage haematopoiesis, transcriptional fidelity to primary tissue, and predictive value for therapeutic responses. Establishing such benchmarks will enable reproducibility, regulatory alignment, and the rational replacement of select animal models, advancing the field toward truly translatable human marrow platforms.

## Figures and Tables

**Table 1:** Observed differences between human and mouse haematopoiesis.

Feature	Human	Mouse	Refs
*HSC cycling speed (in vivo)*	175-350 days	30-50 days	(33–36)
*Clonality in aged* *bone marrow*	Clonal expansion with decreased clonal diversity	Maintained clonal diversity	(37)
*Lineage output of aged bone marrow*	Increased myeloid progenitor output	More pronounced megakaryocyte/myeloid bias	(37)
*Proportion of* *myeloid cells in bone marrow*	Higher	Lower	(38)
*Adipocyte numbers in* *bone marrow*	Higher	Lower	(39)
*Megakaryotic numbers in bone* *marrow*	Lower	Higher	(39)
